# Seasonality and Species Specificity of Submerged Macrophyte Biomass in Shallow Lakes Under the Influence of Climate Warming and Eutrophication

**DOI:** 10.3389/fpls.2021.678259

**Published:** 2021-10-01

**Authors:** Haoping Wu, Beibei Hao, Hyunbin Jo, Yanpeng Cai

**Affiliations:** ^1^Guangdong Provincial Key Laboratory of Water Quality Improvement and Ecological Restoration for Watersheds, Institute of Environmental and Ecological Engineering, Guangdong University of Technology, Guangzhou, China; ^2^Southern Marine Science and Engineering Guangdong Laboratory (Guangzhou), Guangzhou, China; ^3^Department of Bioscience, Aarhus University, Silkeborg, Denmark; ^4^Institute for Environment and Energy, Pusan National University, Busan, South Korea

**Keywords:** climate warming, eutrophication, submerged macrophyte, *Potamogeton crispus*, *Elodea canadensis*

## Abstract

Climate warming and eutrophication caused by anthropogenic activities strongly affect aquatic ecosystems. Submerged macrophytes usually play a key role in shallow lakes and can maintain a stable clear state. It is extremely important to study the effects of climate warming and eutrophication on the growth of submerged macrophytes in shallow lakes. However, the responses of submerged macrophytes to climate warming and eutrophication are still controversial. Additionally, the understanding of the main pathways impacting submerged macrophytes remains to be clarified. In addition, the influence of seasonality on the growth responses of submerged macrophytes to climate warming and eutrophication requires further elucidation. In this study, we conducted a series of mesocosm experiments with four replicates across four seasons to study the effects of rising temperature and nutrient enrichment on the biomass of two submerged macrophytes, *Potamogeton crispus* and *Elodea canadensis*. Our results demonstrated the seasonality and species specificity of plant biomass under the influence of climate warming and eutrophication, as well as the main explanatory factors in each season. Consistent with the seasonal results, the overall results showed that *E. canadensis* biomass was directly increased by rising temperature rather than by nutrient enrichment. Conversely, the overall results showed that *P. crispus* biomass was indirectly reduced by phosphorus enrichment *via* the strengthening of competition among primary producers. Distinct physiological and morphological traits may induce species-specific responses of submerged macrophytes to climate warming and eutrophication, indicating that further research should take interspecies differences into account.

## Introduction

Because of anthropogenic activities, rapid changes, such as climate change and pollution have exerted strong stress on aquatic ecosystems globally (IPCC, 2014; Steffen et al., 2015). Climate warming resulting from accelerated urbanization has increased the water temperature of the hydrosphere over recent decades, and it is expected to continue to increase over this century (IPCC, 2014). On the other hand, the rapid development of agriculture and industrialization has led to massive inputs of nutrients, especially nitrogen (N) and phosphorus (P), in aquatic environments (Tilman et al., 2001). It has been suggested that the alteration of temperature and nutrient availability will change the ecosystem structure and thereafter threaten the ecological functioning of shallow lakes (Liboriussen et al., 2005; Özkan et al., 2010; Kosten et al., 2012; Hao et al., 2020). Submerged macrophytes are an important component in aquatic ecosystems and usually play a vital role in the ecological functioning of shallow lakes (Jeppesen et al., 2012; Hao et al., 2017). It is well-known that submerged macrophytes can maintain clear water by regulating nutrient retention and cycling in aquatic ecosystems (Jeppesen et al., 2012; Wu et al., 2021).

However, the growth of submerged macrophytes is sensitive to climate warming and eutrophication. Increasing temperatures within an optimal range can usually enhance the activity of enzymes and, therefore, promote photosynthesis in submerged macrophytes (Olesen and Madsen, 2000; Riis et al., 2012). Simultaneously, rising temperatures can accelerate dissimilation processes, such as respiration and senescence, and when dissimilation overtakes assimilation, a decline in the biomass of submerged macrophytes occurs (Lee et al., 2007; Hao et al., 2018). Although submerged macrophytes can take up N and P from the water column, satisfy metabolic demand, and stimulate plant growth (Elser et al., 2007; Kaldy, 2014), excessive nutrient loading in water may lead to algal blooms and then shift clear water to a stable turbid state; ultimately, competition with phytoplankton may induce the degradation of macrophytes (Jeppesen et al., 2012; Zhang et al., 2020). Indeed, both climate change and eutrophication usually arise simultaneously in natural systems (Cross et al., 2015). Previous studies have suggested that climate warming and eutrophication can interact to affect the biomass of aquatic plants (Cross et al., 2015; Zhang et al., 2020), while several studies have argued that the effects of temperature and nutrients on the growth of submerged macrophytes are not synergistic (Kaldy, 2014; Trochine et al., 2014). Thus, the effects of climate warming and eutrophication on the biomass of submerged macrophytes remain to be clarified.

Climate warming and eutrophication can directly affect the growth of submerged macrophytes. For example, Zhang et al. (2016, 2019) found that temperature and nutrient availability could significantly affect the growth of submerged macrophytes by altering individual ecological stoichiometry. However, Hao et al. (2018) and Jones et al. (2002) suggested that temperature and nutrients might regulate the growth of submerged macrophytes through the indirect pathway by which rising temperature and nutrient enrichment could induce environmental stress and then cause a decline in the biomass of submerged macrophytes. Furthermore, rising temperature and nutrient enrichment could change abiotic variables, such as dissolved oxygen (DO) and pH, and, thus, influence submerged macrophytes, because DO is necessary for respiration, and pH can determine the availability of inorganic carbon (C) for submerged macrophytes (Jones et al., 2002; Zhang et al., 2015; Dülger et al., 2017; Hao et al., 2020). On the other hand, temperature and nutrients could affect submerged macrophytes through effects on biotic variables, such as the biomass of phytoplankton, periphyton, and zooplankton, since stress from the same trophic level and higher trophic levels may threaten the growth of submerged macrophytes (Jones et al., 2002; Ventura et al., 2008; Hao et al., 2018; Matsuzaki et al., 2018; Yuan and Pollard, 2018). Previous studies have been commonly limited to either direct pathways or indirect pathways but have failed to determine the main pathway impacting the growth of submerged macrophytes, as well as the relative contributions of biotic and abiotic variables to the variation in the biomass of submerged macrophytes.

Taking the life history of an organism into account, seasonality may play a key role in the growth of submerged macrophytes under the influence of rising temperature and nutrient enrichment (Staehr and Sand-Jensen, 2006; Trochine et al., 2014; Zhang et al., 2019; Fu et al., 2020; Hao et al., 2020). For example, Riis et al. (2012) suggested that some submerged macrophyte species were controlled by high summer temperatures rather than low winter temperatures. Hao et al. (2020) found that low temperatures and lower light availability in winter created a harsh environment for primary producers and stressed changes in explanatory variables. However, the experimental cycle in similar studies has usually lasted only dozens of days or one season (Olesen and Madsen, 2000; Riis et al., 2012; Velthuis et al., 2017; Hao et al., 2018; Zhang et al., 2020), which has precluded observation of the response of submerged macrophytes to rising temperature and nutrient enrichment based on the seasonal scale, as well as determination of the main explanatory variables in each season.

**Figure 1:**
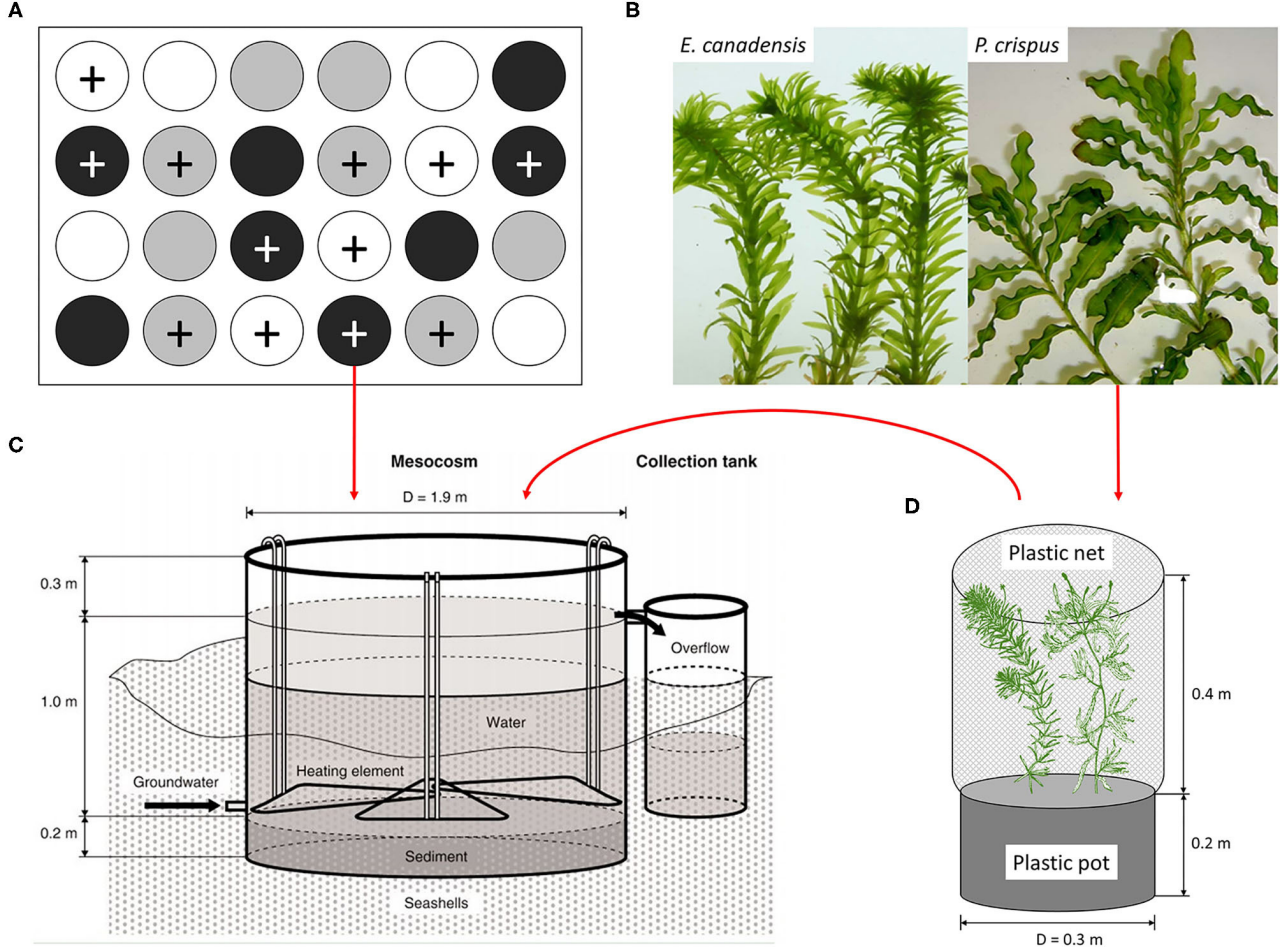
**(A)** Diagram of the experimental design, **(B)** the experimental plant materials, **(C)** the mesocosm tank equipped with a flow-through system, and **(D)** the plastic pot planted with two individual plants, based on Liboriussen et al. (2005) and Trochine et al. (2014). The experimental design includes 24 tanks with two nutrient levels, unenriched (no symbol) and enriched (+ symbol), and three temperature scenarios, ambient (white), warming (gray), and enhanced warming (black).

Here, we established four mesocosm experiments in four seasons to study the effects of climate warming and eutrophication on the growth of submerged macrophytes at the seasonal scale. Two nutrient levels (enriched and unenriched) varied with three temperature scenarios (ambient, IPCC A2, and A2 + 50%) were applied to our mesocosm system. To better simulate the natural system and seasonality, the temperature gradient was set up on the basis of ambient temperature, and the increment in modeled temperature above ambient temperature in each season was different following IPCC climate scenario A2; therefore, the dynamics of modeled temperature varied with the ambient temperature in each season. Two dominant species of submerged macrophytes colonized naturally in most of the unenriched tanks, namely, *Potamogeton crispus* and *Elodea canadensis*, were selected as target species for our experiment. *P. crispus* is a Euro-Asiatic species, while *E. canadensis* originated from North America and has developed into the most common invasive species in Europe (Nichols and Shaw, 1986; Bolduan et al., 1994; Pilon et al., 2003; de Winton et al., 2009; Hussner, 2012; Riis et al., 2012; Wang et al., 2013; Zhang et al., 2015). Life history and morphological structure are conspicuously distinct between *P. crispus* and *E. canadensis* (Nichols and Shaw, 1986; Hao et al., 2020). Specifically, *P. crispus* has a thick branching stem accompanied with sparse strip leaves, while *E. canadensis* has a thin branching stem accompanied by dense whorled leaves (Figure 1). Additionally, *P. crispus* germinates in winter and blooms in summer, whereas *E. canadensis* grows faster in summer and spreads in autumn. Both two species have the same growth form (elodeid) and are equivalent competitors possess multiple adaptations, such as rapid propagation ability, opportunistic nature for nutrient acquisition, high tolerance for low temperature, high photosynthetic efficiency, which allow them to maintain stable coexistence in various regions (Nichols and Shaw, 1986; Vestergaard and Sand-Jensen, 2000; Burson et al., 2019). We hypothesized that the biomass of submerged macrophytes would be directly and indirectly affected by elevated temperature and nutrient enrichment, and that seasonality would contribute to the variance in plant biomass to a certain extent.

## Materials and Methods

### Mesocosm Establishment

A mesocosm system, including 24 experimental tanks (Figure 1; diameter: 1.9 m, water depth: 1 m), was set up in a lowland valley in Central Jutland, Denmark (56° 14′N, 9°31′E) and has been running continuously since August 2003 (Liboriussen et al., 2005). These mesocosms simulate natural shallow lake ecosystems and have been used to study the long-term impacts of climate warming and eutrophication. Each tank was equipped with a flow-through system that automatically adds tap water and removes excess surface water. An experimental design consisting of three temperature scenarios and two nutrient levels (four replicates for each treatment) was applied to the mesocosm system. Briefly, eight tanks were heated according to IPCC climate scenario A2 (warming), eight tanks were heated according to A2 + 50% (enhanced warming), and eight tanks were unheated (ambient) and used as a reference. The IPCC climate scenarios A2 and A2 + 50% were formulated based on regional downscaling (over five 25 km × 25 km grid cells) to the region and applied as the low- and high-warming scenarios, respectively. According to the IPCC, warming was calculated as the average air temperature increase in one particular month with respect to a reference period (1961–1990) and the modeled temperatures in the same month from 2071 to 2100 (Liboriussen et al., 2005). Additionally, climate scenario A2 takes seasonal variation into account, and the modeled temperatures are 2.74, 3.84, 3.76, and 2.76°C higher than the ambient temperatures in May, August, November, and February, respectively. Although IPCC scenarios are based on air temperature, water temperature is used as a surrogate for air temperature in our study, because air and water temperature have tended to be linearly correlated in many previous studies (Liboriussen et al., 2005; Caissie, 2006; Li et al., 2016). In fact, projected changes in aquatic environments under climate change conditions have been based on the strong correlation between water and air temperature, especially for small non-groundwater-dominated water bodies (Caissie, 2006). To mimic eutrophication, Ca(NO_3_)_2_ and Na_2_HPO_4_ solutions were added to 12 tanks weekly, maintaining a constant loading of 538 mg N and 54 mg P per tank each week. The other 12 tanks were treated as references, receiving nutrient input only from the tap water and remaining in an unenriched state (51–71 μg N L^−1^ and 2–20 μg P L^−1^).

### Experimental Design

This study was performed with the mesocosm system described above (Figure 1). The mesocosm system has been running continuously for 15 years before the initiation of our experiment, ensuring the reliability of the experiment, because the aquatic organisms in the mesocosm system may have evolved and adapted to the current conditions. To examine the effects of climate warming and eutrophication on the growth of submerged macrophytes, shoots of *P. crispus* and *E. canadensis* (Figure 1) were collected from the mesocosm system to conduct the experiment. For the purpose of distinguishing the effect of seasonality, four independent sub-experiments were conducted in May (spring), August (summer), November (autumn), and February (winter), and each sub-experiment lasted for 3 weeks.

The process for each sub-experiment was as follows: each shoot for a particular species was collected from the same stock plant to ensure the same growth stage, thoroughly rinsed to remove the surficial attachment, and weighed to ensure the same initial biomass (±0.05 g). One *P. crispus* and one *E. canadensis* were planted together in the same plastic pot and filled with sediment collected from the same mud and thereafter covered with a layer of 2-cm pure sand to prevent nutrient exchange between the sediment and the surface water (Figure 1). A plastic net was fixed around the edge of the plastic pot to protect the plants and associated epiphytic algae from snail herbivory. Subsequently, each plastic pot was randomly transferred to one tank and hung in the center of the tank at a depth of 25 cm. In all, 24 plastic pots planted with two kinds of shoots were hung in 24 tanks, corresponding to three temperature scenarios and two nutrient levels (four replicates for each treatment) (Figure 1).

### Sampling and Parameter Detection

The entire plant was harvested from the tank for each submerged macrophyte, and the sediment was carefully removed *in situ*. Approximately 1 L of surface water was sampled from each tank before plant collection. All the samples were taken back to the laboratory for further examination.

All the biotic parameters were measured in the laboratory. Initially, each individual was rinsed thoroughly with distilled water, and the rinsing water was collected to examine periphyton attached to the surface of the leaves. Next, each whole plant was dried with absorbent paper and weighed to determine fresh biomass. Subsequently, each plant sample was flattened and placed under a lens, and a digital photograph was taken. After that, the photographs were scanned using the ImageJ software, and the scanned areas were multiplied by 2 to estimate the surface areas of the plant leaves. In addition, each rinsing water sample was adjusted to a volume of 1,000 ml and filtered through a GF/C filter membrane (0.45 μm; Whatman, Maidstone, United Kingdom), and the residue in the filter was placed in a 10-ml 95% ethanol solution to extract photosynthetic pigments and measure chlorophyll *a* (Chl-*a*) content *via* a spectrophotometer (UV-1800; Shimadzu, Jena, Germany). Consequently, periphyton Chl-*a* was estimated as the mass of Chl-*a* per leaf area of each plant (μg/cm^2^) (Hao et al., 2020). Similarly, each acquired 500-ml surface water sample was filtered and extracted, and then phytoplankton Chl-*a* was measured. The identification and enumeration of zooplankton were performed under an inverted microscope.

Several abiotic environmental factors, such as water temperature (Temp), DO, electrical conductivity, and pH, were determined *in situ* using a water quality meter (YSI 6920; YSI Inc., Yellow Springs, OH, United States) before sampling, while the concentrations of total nitrogen (TN) and total phosphorus (TP) in water were determined using a UV-visible spectrophotometer (UV-1800; Shimadzu, Jena, Germany) in the laboratory.

### Statistical Analysis

After the normality and homoscedasticity of the data were confirmed by the Shapiro-Wilk test, two-way ANOVA and *post-hoc* tests were performed to examine the effects of temperature and nutrients, and their interactions on the biomass of submerged macrophyte during each season. In addition, the Scheirer–Ray-Hare test was performed to examine the responses of environmental variables to temperature and nutrients. Thereafter, the pairwise Wilcoxon rank-sum test with Bonferroni–Holm adjustment was performed for *post-hoc* pairwise comparisons. In addition, the main factors explaining the variance in plant biomass in each season were analyzed by redundancy analysis (RDA). After Spearman correlation analysis, a structural equation model (SEM) was used to explore the direct and indirect pathways by which temperature and nutrients affected the growth of submerged macrophytes. The Shapiro–Wilk test, two-way ANOVA, and Spearman correlation analysis were performed with SPSS (Version 20.0; SPSS Inc., Chicago, IL, United States), while the Scheirer-Ray-Hare test, pairwise Wilcoxon rank-sum test, and RDA were performed in R (version 3.6.1) with the rcompanion and vegan packages. SEM was performed with AMOS (Version 21.0, IBM SPSS Amos; SPSS Inc., Chicago, IL, United States).

## Results

### Responses of Plant Biomass to Temperature and Nutrients in Each Season

According to the results of two-way ANOVA, significant effects of temperature could be observed on *E. canadensis* biomass in spring (*p* = 0.012), summer (*p* = 0.001), and autumn (*p* = 0.043), and on *P. crispus* biomass only in spring (*p* = 0.034). On the other hand, the only significant effect of nutrients occurred on *P. crispus* biomass in autumn (*p* = 0.043). Furthermore, the interaction of temperature and nutrients significantly affected *E. canadensis* biomass in spring (*p* = 0.012), whereas neither temperature nor nutrients significantly affected the biomass of either species in winter.

**Figure 2:**
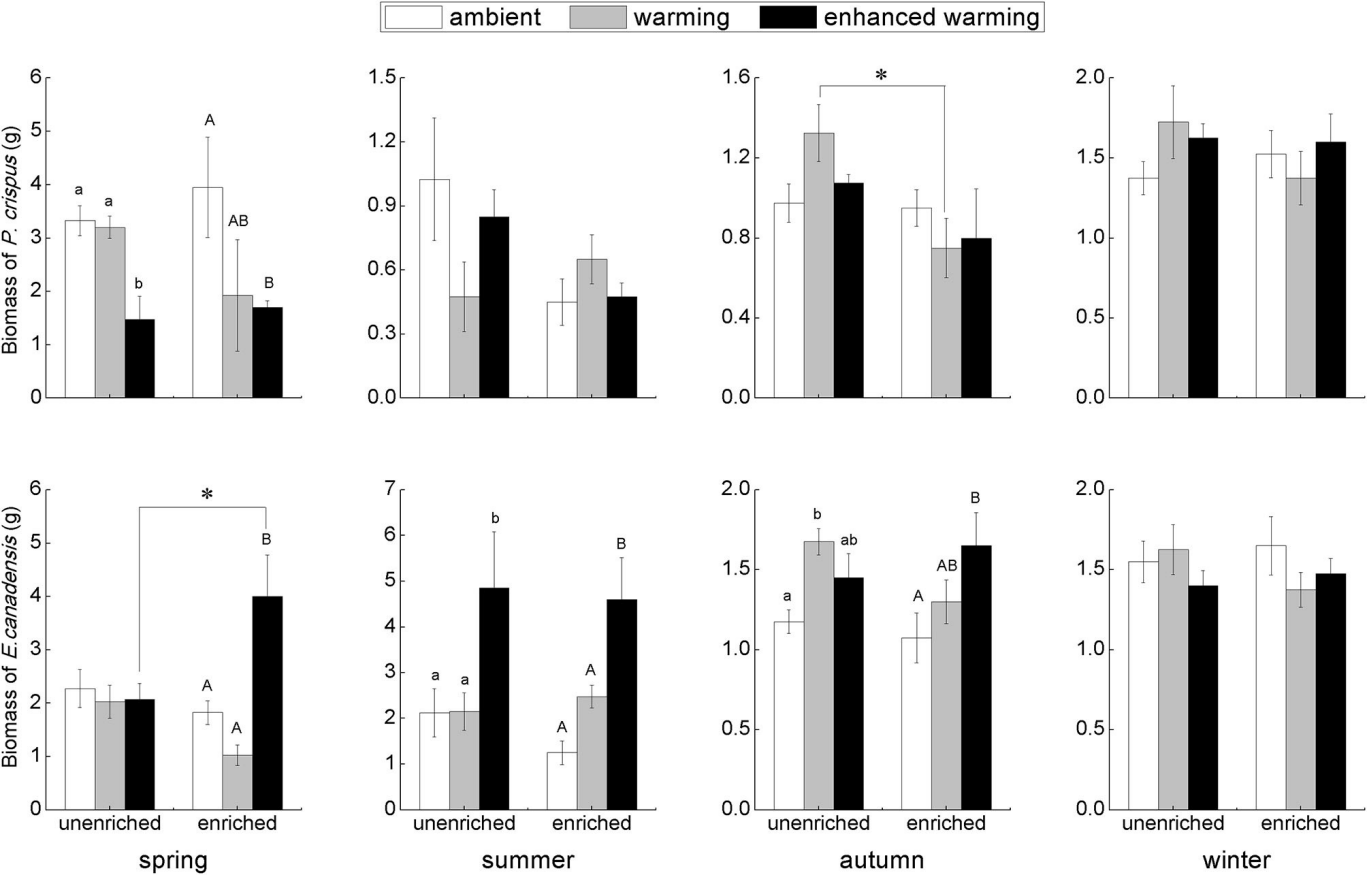
The biomass (mean ± SE) of submerged macrophytes at different levels of temperature and nutrients in each season. Different letters indicate significant differences (*p* < 0.05) among the three temperature scenarios. The asterisks above the bars indicate significant differences (*p* < 0.05) between the two nutrient levels.

Our results showed that the biomass of *P. crispus* decreased in the warming scenario, most significantly in the enhanced-warming scenario in spring, but that the responses of *P. crispus* biomass to the temperature gradients were ambiguous in other seasons (Figure 2). In contrast, *E. canadensis* biomass significantly increased in the warming scenario, especially in the enhanced-warming scenario, in most seasons except winter (Figure 2). In summer and autumn, *E. canadensis* biomass increased significantly with temperature across the two nutrient levels (Figure 2). Based on the nutrient levels, *P. crispus* biomass showed a decreasing tendency under the effect of nutrient enrichment in most seasons except in winter, as was especially evident with the significant decline that occurred in the warming scenario in autumn (Figure 2). Despite the significantly higher biomass of *E. canadensis* in enriched relative to unenriched conditions in the enhanced-warming scenario in spring, nutrients generally had little effect on *E. canadensis* biomass in most cases (Figure 2).

### Responses of Environmental Factors to Temperature and Nutrients in Each Season

**Figure 3:**
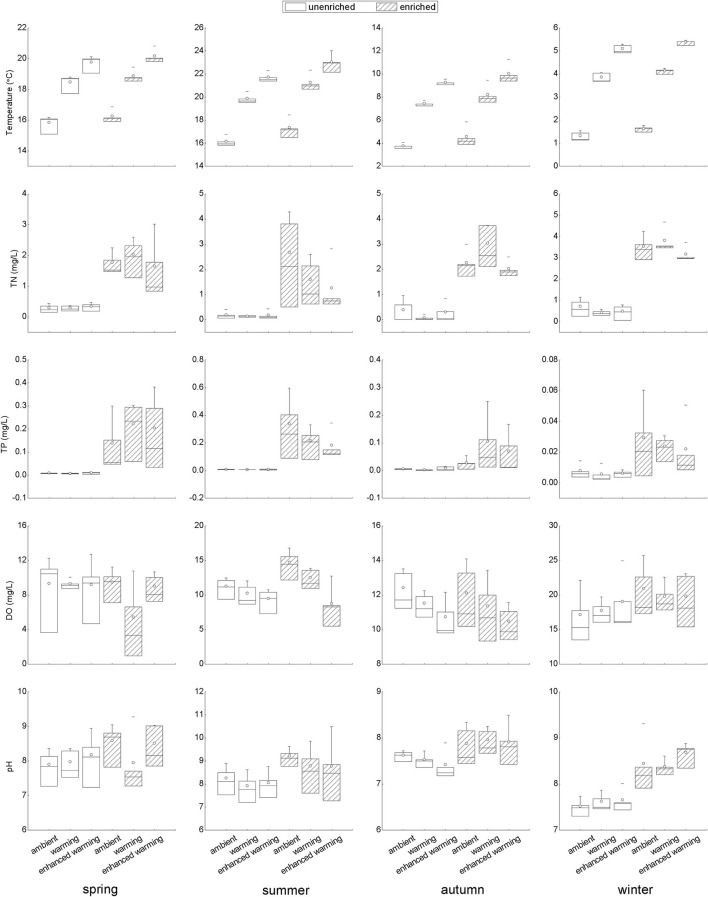
Variation in temperature and nutrient concentrations and responses of abiotic variables to temperature and nutrients in each season. TN, total nitrogen in water; TP, total phosphorus in water; DO, dissolved oxygen.

The temperature gradients and levels of nutrients, including TN and TP, clearly fit the experimental design well (Figure 3). Of several abiotic environmental factors, DO showed a decreasing tendency with increasing temperature in summer and autumn; in particular, there was a significant difference between the ambient and enhanced-warming scenarios (Figure 3). In contrast, the variation in pH might have been affected by nutrient enrichment, as higher pH was observed in enriched treatments in each season, most significantly in winter (Figure 3).

**Figure 4:**
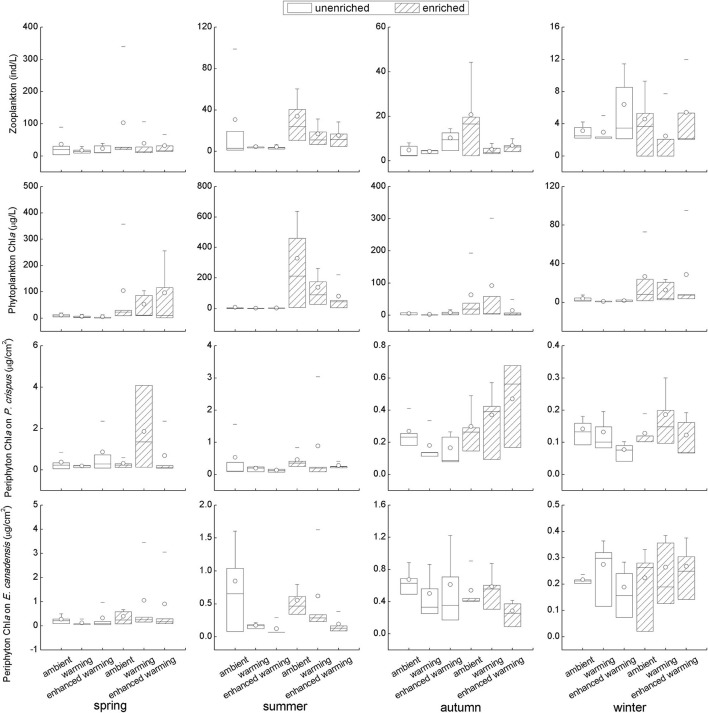
Responses of biotic variables to temperature and nutrients in each season. It should be noted that the vertical scale in each figure was different among seasons.

Although some results with strong variability seemed statistically unsupported, it was clear that the biotic environmental factors, including the biomass of zooplankton, phytoplankton, and periphyton, were generally higher in the enriched group than in the unenriched group on the basis of nutrient level; in particular, a significant increase in each biotic variable in the enriched treatment was found in summer (Figure 4). Furthermore, an opposing tendency was apparent between phytoplankton and periphyton; for example, the increase in phytoplankton was accompanied by a decline in periphyton in spring and summer and vice versa in autumn and winter (Figure 4). Despite the significantly higher biomass of periphyton on *E. canadensis* in the ambient than in the enhanced-warming scenario in summer, and the tendency of zooplankton to decrease with increasing temperature in enriched tanks, the overall trends of biotic variables in response to temperature remained ambiguous (Figure 4). Specifically, intense declines in the biomass of zooplankton, phytoplankton, and periphyton were observed in winter relative to their levels in the other seasons (Figure 4).

### Key Environmental Factors Affecting Plant Biomass in Each Season

**Figure 5:**
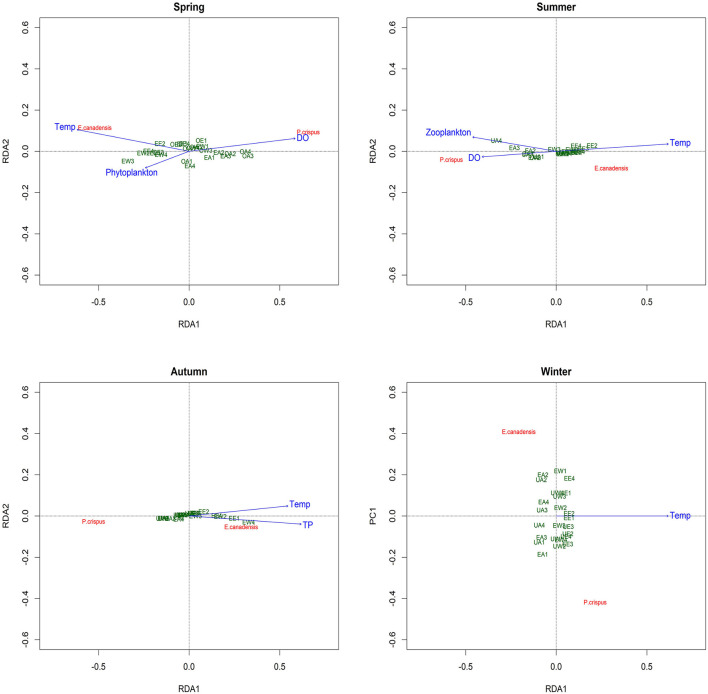
Redundancy analysis (RDA) plot showing the analysis of the correlations between the biomass of submerged macrophytes and main explanatory factors in each season. Temp, temperature; DO, dissolved oxygen; TP, total phosphorus in water; UA, unenriched and ambient; UW, unenriched and warming; UE, unenriched and enhanced warming; EA, enriched and ambient; EW, enriched and warming; EE, enriched and enhanced warming.

The RDA revealed the main explanatory factors contributing to the variance in submerged macrophyte biomass in each season except winter (Figure 5). In particular, temperature and DO were significant factors explaining 11 and 8%, respectively, of the variance (adjusted R^2^) in spring; the former was positively correlated with *E. canadensis*, while the latter was positively correlated with *P. crispus*. Despite the nonsignificant contribution (3%) to the variance in spring, phytoplankton showed a notable negative correlation with *P. crispus*. Additionally, temperature and DO were both significant explanatory variables accounting for 22 and 16%, respectively, of the variance in summer. Moreover, a significant contribution (7%) by zooplankton to the variance and a positive relationship with *P. crispus* biomass could be observed in summer. In autumn, TP was the only significant explanatory factor, accounting for 10% of the variance, while temperature exhibited a noticeable contribution (4%) to the variance, and both of them were positively correlated with *E. canadensis* but negatively correlated with *P. crispus*. Unfortunately, no significant explanatory factor could be found in winter.

### Pathways by Which Temperature and Nutrients Affect Plant Biomass

**Figure 6:**
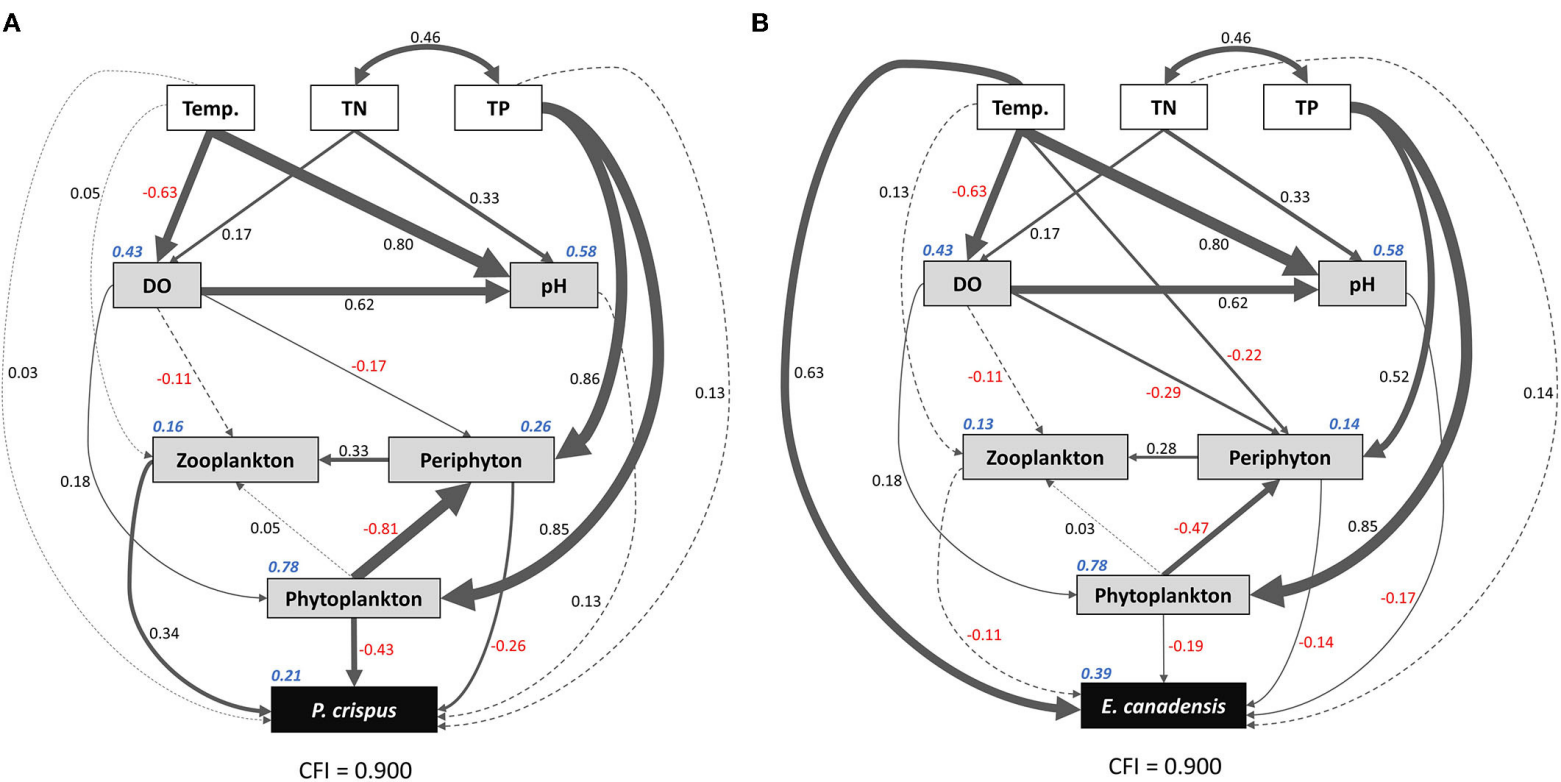
Structural equation model (SEM) showing the direct and indirect pathways by which temperature and nutrients affected the biomass of *P. crispus* **(A)** and *E. canadensis* **(B)**. Single-headed arrows represent unidirectional causal relationships, and double-headed arrows represent non-causal covariances. Solid lines indicate significant (*p* < 0.05) or marginally significant (*p* < 0.1) effects, and dashed lines indicate non-significant effects. Numbers adjacent to the lines are standardized path coefficients, analogous to relative regression weights and indicative of the effect sizes of the corresponding relationships. The thickness of the arrows is proportional to the magnitudes of the standardized path coefficients or covariation coefficients. Numbers in italics adjacent to the boxes provide the *R*^2^ values and denote the proportions of submerged macrophyte biomass explained by these parameters. Overall goodness-of-fit tests (CFIs) are shown at the bottom of each figure. Temp., temperature; TN, total nitrogen in water; TP, total phosphorus in water; DO, dissolved oxygen.

Based on the results of the Spearman correlation analysis, the variables that were significantly correlated with each other were selected to run the SEM. Here, the experimental treatments, including temperature, TN, and TP, were selected as promising explanatory factors; environmental variables, including abiotic and biotic factors, were selected as intermediate factors; and biomass values of submerged macrophytes were selected as the target variables. Consequently, the comparative fit index (CFI) value = 0.9 indicated that the final model was acceptable. The *R*^2^ values were 0.21 and 0.39 for *P. crispus* biomass and *E. canadensis* biomass, respectively, implying a considerable contribution of other factors to variation in the target variables (Figure 6).

The results of the SEM analysis illustrated the direct and indirect pathways by which temperature and nutrients affected the biomass of submerged macrophytes (Figure 6). It was clear that temperature exerted a significant direct impact on *E. canadensis* biomass (0.63) and abiotic variables such as DO (−0.63) and pH (0.8) but not on *P. crispus* biomass or most of the biotic variables such as phytoplankton, periphyton, and zooplankton (Figure 6). Although there was a little direct effect of nutrients on plant biomass, we found that TN could significantly and directly increase abiotic variables, including DO (0.17) and pH (0.33), while TP could significantly and directly increase biotic variables, including the biomass of phytoplankton (0.85), the biomass of periphyton on *P. crispus* (0.86), and biomass of periphyton on *E. canadensis* (0.52) (Figure 6). When the direct and indirect effects were combined into total effects, we found that *E. canadensis* biomass was strongly increased by rising temperature directly (Figure 6, Table 1). In contrast, *P. crispus* biomass was reduced directly by phytoplankton and periphyton and indirectly by TP but was increased directly by zooplankton (Figure 6, Table 1). Moreover, a positive relationship between zooplankton and periphyton and a negative relationship between phytoplankton and periphyton were demonstrated by the SEM (Figure 6).

**Table 1:** Standardized total, direct, and indirect effects of environmental factors on the biomass of submerged macrophytes in the SEM diagram.

	***P. crispus***			***E. canadensis***		
	**Direct effect**	**Indirect effect**	**Total effect**	**Direct effect**	**Indirect effect**	**Total effect**
Temp.	0.027	0.112	0.139	0.626	−0.072	0.554
TN	–	0.045	0.045	0.126	−0.067	0.059
TP	0.134	−0.377	−0.243	–	−0.186	−0.186
DO	–	0.014	0.014	–	−0.064	−0.064
pH	0.128	–	0.128	−0.170	–	−0.170
Zooplankton	0.341	–	0.341	−0.105	–	−0.105
Phytoplankton	−0.429	0.133	−0.295	−0.190	0.079	−0.112
Periphyton	−0.258	0.112	−0.146	−0.145	−0.029	−0.174

*Direct effects are equal to the standardized path coefficients presented in Figure 6. Indirect effects refer to the mathematical products of all possible paths. Total effects are the sums of direct and indirect effects. Temp., temperature; TN, total nitrogen in water; TP, total phosphorus in water; DO, dissolved oxygen*.

## Discussion

Even though there were only four replications, our study compiled a large number of samples and demonstrated noticeable seasonality and species specificity in the growth of submerged macrophytes and in the variation of environmental factors under the influence of climate warming and nutrient enrichment, consistent with previous studies (Staehr and Sand-Jensen, 2006; Trochine et al., 2014; Zhang et al., 2019; Fu et al., 2020; Hao et al., 2020). Here, a significant negative effect of temperature on plant growth was observed only in *P. crispus* in spring, while a significant positive effect of temperature on plant growth appeared in *E. canadensis* in each season except winter (Figure 2). Moreover, a significant increase in *E. canadensis* biomass occurred with nutrient enrichment coupled with the enhanced-warming scenario in spring, indicating that the interaction of climate warming and nutrient enrichment only increased *E. canadensis* growth during spring (Figure 2). Similar results could be observed for several abiotic and biotic variables. For example, DO declined with increasing temperature in all the seasons except winter (Figure 3). In addition, the responses of sestonic and epiphytic algal biomass to nutrient enrichment were significant only in summer (Figure 4). The seasonality of plant biomass and environmental variables imply that the effects of climate warming and eutrophication on the aquatic ecosystems were sensitive to season.

Although the main factors affecting the growth of submerged macrophytes varied among seasons, the effects of temperature were sustained across the four seasons, suggesting the vital role climate warming played in the growth of submerged macrophytes (Figure 5). Such a strengthening effect of climate warming on the growth of *E. Canadensis* may be attributed to the stimulation of plant metabolism by high temperature, which is consistent with findings for the same species in previous studies (Olesen and Madsen, 2000; Riis et al., 2012; Zhang et al., 2015) and prevalent in many kinds of submerged macrophytes, such as *Callitriche cophocarpa, Zostera marina, Elodea nuttallii, Vallisneria spiralis*, and *Potamogeton lucens* (Olesen and Madsen, 2000; Kaldy, 2014; Zhang et al., 2019). In contrast, the restraining effect of high temperature on *P. crispus* during its growing season agrees with the results of a similar experiment conducted by Hao et al. (2018) in China and suggests that warming might accelerate the life cycle of *P. crispus* and increase respiration more than production, ultimately causing a decline in plant biomass during the growing period (Lee et al., 2007; Zhang et al., 2016; Hao et al., 2018). It is well-known that rising temperatures could significantly decrease DO concentrations in water because of enhanced oxygen consumption by intensive respiration from plant metabolism or from decomposition (Veraart et al., 2011; Zhang et al., 2015; Hao et al., 2020). A significant negative response of DO to rising temperature is confirmed in our study (Figures 3, 6). The positive effect of DO on *P. crispus* in spring and summer (Figure 5) might be ascribed to the high demand for oxygen for enhanced respiration. Considering that the abundance of phytoplankton and zooplankton rose sharply in spring and summer (Figure 3), the negative effect of phytoplankton and the positive effect of zooplankton on *P. crispus* biomass were not surprising (Figures 5, 6). Interestingly, in summer, the DO in enriched tanks in the enhanced-warming scenario was lowest (Figure 3), and concomitant lower *P. crispus* biomass and all biotic variables, but higher *E. canadensis* biomass could be found (Figures 2, 4). This phenomenon probably provides speculation on how the two species and their ecosystems would vary in a scenario where temperature rises dramatically with consequent hypoxia and eutrophication.

However, the decline in microorganisms in cold seasons, namely, autumn and winter, may diminish the influence of biotic factors on submerged macrophytes, and the main contribution may be replaced by those of abiotic factors such as temperature or nutrients (Menge and Sutherland, 1987; Hao et al., 2020). In our study, the decline in *P. crispus* in the enriched treatment (Figure 2) in autumn was mainly induced by TP enrichment (Figure 5). The N:P ratio has been successfully used to predict the nature of community nutrient limitation in multiple aquatic ecosystems (Koerselman and Meuleman, 1996). In this study, an average N:P ratio below 6.1 indicated P overloading rather than P limitation for the plants (Wang et al., 2013). The supply of additional P at low P concentrations was conducive to the photosynthesis of *P. crispus*, while a high P concentration might negatively impact photosynthesis because of an imbalance in nutrient supply (Majerowicz and Kerbauy, 2002; Wang et al., 2013). Low P supply has been found to promote plant starch synthesis, leading to the release of inorganic P into the cytoplasm from sugar-binding materials and maintenance of a constant cytoplasmic inorganic P concentration (Franco-Zorrilla et al., 2005). However, increasing water P levels might result in a lower capacity for starch synthesis in *P. crispus* and, thus, reduce inorganic P release into the cytoplasm (Wang et al., 2013). Owing to reduced solar radiation and persistent ice cover, winter is usually characterized by low temperature and limited light availability, which creates an unfavorable habitat for aquatic primary producers (Gustina and Hoffmann, 2000; Hao et al., 2020). Therefore, the lower ambient temperature (average 1.5°C in February vs. 16.8°C in August) and continuous ice cover on most of the experimental tanks in winter in our study would be expected to result in a great decline in plankton and periphyton (Figures 3, 4). This may help explain why the environmental factors showed no significant effects accounting for the variance in plant biomass during winter, despite the apparent influence of temperature (Figure 5).

When variation from the four seasons was synthesized, the overall biomass of *E. canadensis* was positively affected primarily by temperature rather than nutrients, consistent with the results at the seasonal scale (Figures 5, 6, Table 1). This result is unsurprising because the optimum temperature range for photosynthesis of submerged macrophytes is between 25 and 35°C when reviewing data across a series of submerged macrophyte species (Santamaría and van Vierssen, 1997). It has been widely confirmed that rising temperatures could enhance the growth of multiple aquatic plant species within their favorable temperature ranges (Madsen and Brix, 1997; Kaldy, 2014; Velthuis et al., 2017; Zhang et al., 2019). Aquatic plants can acclimate to thermal variation by phenotypic modifications of physiology and morphology (Barko et al., 1982; Madsen and Brix, 1997; Olesen and Madsen, 2000; Pilon and Santamaría, 2002; Zhang et al., 2015). For example, Olesen and Madsen (2000) and Riis et al. (2012) found that the relative growth rate and photosynthetic capacity of *E. canadensis* were enhanced by high temperatures. In addition, Pilon and Santamaría (2002) and Zhang et al. (2015) observed that the competitive traits of *Potamogeton pectinatus* and *E. canadensis*, such as leaf area, stem length, and root:shoot ratio, increased with increasing temperature, thereby promoting the growth and biomass of whole plants.

According to the hypothesis of temperature-plant physiology and enhanced nutrient-use efficiency (Reich and Oleksyn, 2004; An et al., 2005; De Senerpont Domis et al., 2014), elevated temperature would lead to an increased plant C:N ratio. As the temperature increases, on the one hand, aquatic plants invest fewer nutrients per mass of carbon to produce proteins to sustain biochemical reactions (Zhang et al., 2016, 2019). On the other hand, aquatic plants grow longer and accumulate additional biomass to develop their competitive capacity in response to rising temperatures, thereby diluting plant N and P contents (Velthuis et al., 2017; Zhang et al., 2020). In this study, the pH varied from 7 to 10 (Figure 3), which indicates that the major C source for the submerged macrophytes was bicarbonate (${\text{HCO}}_{3}^{-}$) (Maberly and Gontero, 2017; Zhang et al., 2020). Previous experiments conducted in the same mesocosm system have reported that the alkalinity in each tank was always above 1 meq L^−1^ (Ventura et al., 2008; Zhang et al., 2015), signifying that the growth of *E. canadensis* is probably not limited by inorganic C availability (Vestergaard and Sand-Jensen, 2000; Zhang et al., 2019). Furthermore, nutrient enrichment compensated for the nutrient dilution effect, which was reflected by the interaction effect of rising temperature and nutrient enrichment on the growth of *E. canadensis* in spring (Figure 2).

For *P. crispus*, based on direct pathways, the overall variation in plant biomass was primarily affected by biotic factors rather than abiotic factors; meanwhile, based on the indirect pathways, it was primarily affected by P enrichment rather than temperature (Figure 6, Table 1). Numerous studies have revealed tight linkages among plankton, periphyton, and plants in aquatic ecosystems (Jones et al., 2002; Gyllström et al., 2005; Ventura et al., 2008; Özkan et al., 2010; Trochine et al., 2014; Hao et al., 2018, 2020; Matsuzaki et al., 2018; Yuan and Pollard, 2018; Zhang et al., 2020). The significant negative relationships between phytoplankton, periphyton, and plants in our study (Figure 6) indicate an intense direct competition among epiphytes, sestonic producers, and macrophytes, which is prevalent across the findings of a variety of studies (Jones et al., 2002; Ventura et al., 2008; Özkan et al., 2010; Trochine et al., 2014; Hao et al., 2020; Zhang et al., 2020). Phytoplankton, periphyton, and submerged macrophytes differ in their uptake of nutrients. Algae can take up nutrients from the water column even more efficiently than aquatic plants (Zhang et al., 2020). Similarly, free-floating algae can gain access to available nutrients in the water column more easily than adherent algae, which are constrained by the effects of the shape and boundary layer of biofilm (Trochine et al., 2014). In addition, the layer formed by epiphytes acts as a physical barrier to material exchange between plants and the aquatic environment (Jones et al., 2002; Ventura et al., 2008). Consequently, the flourishing of algae resulting from N and P enrichment would aggravate the competition for inorganic C acquisition among primary producers, thereby leading to C starvation of submerged macrophytes (Jones et al., 2002; Ventura et al., 2008; Dülger et al., 2017; Zhang et al., 2020). In addition, the increasing concentration of ${\text{HCO}}_{3}^{-}$ due to the elevated water pH reduced the availability of inorganic C for submerged macrophytes, because the utilization of ${\text{HCO}}_{3}^{-}$ incurred considerable physiological costs (Jones et al., 2002; Dülger et al., 2017). In addition to the C source, the requirement for light availability often shapes the intense competition among primary producers (Jones et al., 2002; Hao et al., 2018; Matsuzaki et al., 2018; Zhang et al., 2020). It has been explained that the increased abundance of epiphytes and sestonic and filamentous algae due to nutrient enrichment can restrict light availability through shading, thereby leading to the loss of submerged macrophytes (Phillips et al., 1978; Hao et al., 2018; Matsuzaki et al., 2018). Based on the annual scale, the probable fundamental cause for the decrease in the biomass of *P. crispus* in our study was external nutrient addition (through competition with increasing algae) rather than rising temperature (Figure 6, Table 1), which agrees with the results of multiple studies (Ventura et al., 2008).

Not only primary producers but also trophic cascades, including top-down and bottom-up control, could affect the growth of submerged macrophytes (Jones et al., 2002; Ventura et al., 2008; Hao et al., 2018; Matsuzaki et al., 2018; Yuan and Pollard, 2018). In fact, nutrient enrichment significantly fueled the boom of algae initially, periphyton then significantly stimulated the development of zooplankton, and zooplankton significantly promoted the growth of *P. crispus* eventually. These sequential linkages in our experiment (Figure 6) indicate that cascading trophic effects had an impact on *P. crispus* through bottom-up control rather than top-down control. Of the zooplankton species in our mesocosm system, many copepods are carnivores or omnivores, while cladocerans and rotifers are grazers that feed on algae (Ventura et al., 2008; Matsuzaki et al., 2018). Carpenter et al. (2001) suggested that bottom-up control in primary production was stronger in lakes with three trophic levels than in lakes with the presence of a fourth trophic level. In addition, Costanza et al. (1993) and Frank et al. (2007) suggested that a shallow depth and temperate climate could promote the dominance of bottom-up control in lakes by accelerating efficient nutrient cycling. Specifically, the edibility of the phytoplankton, as measured by the proportions of edible and inedible algae (usually represented by cyanobacteria) within the phytoplankton assemblage, could determine the importance of bottom-up linkages (Gyllström et al., 2005; Matsuzaki et al., 2018; Yuan and Pollard, 2018). It has been demonstrated that the lack of highly unsaturated fatty acids in cyanobacteria, large size and accompanying gelatinous sheaths of cyanobacterial colonies, and toxins produced by cyanobacteria can threaten the growth of grazers (Wilson et al., 2006; Bednarska and Dawidowicz, 2007; Persson et al., 2007; Yuan and Pollard, 2018). Findings of previous studies have suggested that the dominance of cyanobacteria can be enhanced either by nutrient enrichment or rising temperature, which exerted stress on the bottom-up linkage and ultimately reduced the growth of aquatic plants (Gyllström et al., 2005; Kosten et al., 2012; Trochine et al., 2014; Matsuzaki et al., 2018; Schaum et al., 2018; Yuan and Pollard, 2018). In this study, phytoplankton growth was limited by P loading instead of N loading, and zooplankton was significantly increased by periphyton rather than by phytoplankton, implying that the assemblage of phytoplankton might be primarily composed of N-fixing cyanobacteria; therefore, the edibility of algae has shaped the herbivory strategy of grazers (Figure 6). On the other hand, the decline in zooplankton abundance with increasing temperature in the enriched mesocosms (Figure 4) might be ascribed to the increasing proportion of inedible algae. Thus, both nutrient enrichment and elevated temperature might result in the decline of *P. crispus* biomass by weakening bottom-up control, and this nonsignificant but visible interaction effect of nutrient enrichment and warming on the growth of *P. crispus* was present in autumn (Figure 2).

Although both *E. canadensis* and *P. crispus* are considered tolerant species that are, to some extent, capable of enduring alteration in nutrient loading and temperature (Riis et al., 2012; Zhang et al., 2015, 2016), their performance in response to nutrient enrichment and the rising temperature was conspicuously different in our study (Figure 2). There are three possible explanations for these discrepancies in their behaviors in response to climate warming and eutrophication. First, distinguishing aspects of specific lifecycles may result in differences in tolerance to elevated temperature. *E. canadensis* belongs to the Hydrocharitaceae family (de Winton et al., 2009; Hussner, 2012; Riis et al., 2012), grows faster in summer, and spreads in autumn, implying that elevated temperature would prolong the growing season and thereafter promote the productivity of *E. Canadensis* (Zhang et al., 2015; Hao et al., 2020). In contrast to *E. canadensis, P. crispus* usually germinates in winter, is more tolerant to colder temperatures, and thrives better at low temperatures (Bolduan et al., 1994; Pilon et al., 2003; Wang et al., 2013; Zhang et al., 2015). Second, the morphological complexity of submerged macrophytes can determine the underwater availability of light. Hao et al. (2017) compared the shading effects of several submerged macrophytes featuring morphological complexity gradients and found increasing underwater light attenuation with decreasing morphological complexity. Stronger light attenuation could induce lower light availability, thereby decreasing photosynthesis. *P. crispus* exhibits a simpler morphological complexity than *E. canadensis* (Hao et al., 2020). Thus, the middle and bottom leaves of *P. crispus* may experience low light availability and be sensitive to the turbidity induced by nutrient enrichment. Such a shading effect leading to the decreased survival of *P. crispus* has been demonstrated in a previous study (Hao et al., 2018). Third, the ability to utilize ${\text{HCO}}_{3}^{-}$ efficiently may lead to superiority in competition for inorganic C sources. It has been verified that the mechanism by which *Elodea* and *Potamogeton* utilize ${\text{HCO}}_{3}^{-}$ relies on leaf surface polarity. That is, ${\text{HCO}}_{3}^{-}$is converted into CO_2_ in the cell walls and boundary layer by the activity of H^+^ pumps located at the plasmalemmas of the lower cells *of Elodea* and *Potamogeton*, and released CO_2_ subsequently diffuses into cells (Prins et al., 1982; Madsen et al., 1998). Therefore, *E. canadensis*, which possesses a higher leaf number and wider leaf surface area, may be able to take up ${\text{HCO}}_{3}^{-}$ more efficiently than *P. crispus*. In addition, Sand-Jensen (1983) found that the photosynthetic rate of *P. crispus* was reduced by increasing pH, which could be attributed to the increasing capacity to buffer the efflux of H^+^.

## Conclusion

Taking into account the structuring role of submerged macrophytes in shallow lake ecosystems, it is of great importance to predict the response of submerged macrophytes to climate warming coupled with eutrophication (Jeppesen et al., 2012). In this study, we found that submerged macrophyte biomass showed noticeable seasonality and species specificity in response to climate warming and eutrophication. Additionally, the main explanatory variables accounting for the biomass of submerged macrophytes differed in each season. In addition, a significant interaction effect of temperature and nutrients on the growth of plants only occurred in rare cases. Based on the annual scale, the overall results showed a direct positive effect of temperature rather than nutrient concentrations on *E. canadensis* biomass, which was consistent with the seasonal results. However, the overall results showed that P enrichment negatively affected *P. crispus* biomass indirectly by altering the biotic environmental factors, specifically by strengthening the competition among primary producers. Interestingly, we found that the growth of *P. crispus* was positively affected by a trophic cascade through bottom-up control rather than top-down control. In summary, it can be speculated that continuous climate warming and eutrophication would cause a transition in aquatic plant communities in shallow lakes through selection effects. Species-specific responses of submerged macrophytes to climate warming and eutrophication may be ascribed to their distinct physiological and morphological traits. These conclusions are drawn based on just four experimental replicates. We suggest that further research on the response of submerged vegetation in shallow lakes to climate warming and eutrophication should be conducted from the perspective of the population.

## Data Availability Statement

The raw data supporting the conclusions of this article will be made available by the authors, without undue reservation.

## Author Contributions

HW designed the study, performed the research, collected the samples, analyzed the data, and wrote the manuscript. BH collected the samples and analyzed the data. HJ collected the samples. YC contributed to data analysis and revisions. All authors have reviewed, discussed, agreed to the authorship, and submission of the manuscript for peer review.

## Funding

This study was supported by the National Natural Science Foundation of China (51879007, 52000041, 51979043), the China Scholarship Council (CSC), the China Postdoctoral Science Foundation Grant (2019M662810), the Guangdong Provincial Key Laboratory Project (2019B121203011), the Guangdong Province Basic and Applied Basic Research Fund (2019A1515010378), and the KeySpecial Project for Introduced Talents Team of Southern Marine Science and Engineering Guangdong Laboratory (Guangzhou) (GML2019ZD0403).

## Conflict of Interest

The authors declare that the research was conducted in the absence of any commercial or financial relationships that could be construed as a potential conflict of interest.

## Publisher's Note

All claims expressed in this article are solely those of the authors and do not necessarily represent those of their affiliated organizations, or those of the publisher, the editors and the reviewers. Any product that may be evaluated in this article, or claim that may be made by its manufacturer, is not guaranteed or endorsed by the publisher.
